# Recent advances in understanding cholangiocarcinoma

**DOI:** 10.12703/b/9-15

**Published:** 2020-11-19

**Authors:** Pedro Luiz Serrano Uson Junior, Mansi Arora, James M Bogenberger, Mitesh J Borad

**Affiliations:** 1 Division of Hematology and Medical Oncology, Mayo Clinic, Scottsdale, Arizona, USA; 2 Department of Molecular Medicine, Mayo Clinic, Rochester, Minnesota, USA; 3 Center for Individualized Medicine, Mayo Clinic, Rochester, Minnesota, USA; 4 Mayo Clinic Cancer Center, Mayo Clinic, Phoenix, Arizona, USA; 5 Hospital Israelita Albert Einstein, Sao Paulo, Brazil

**Keywords:** Cholangiocarcinoma, biliary tract cancer, FGFR2, IDH1, PD-1

## Abstract

The definition of cholangiocarcinoma (CCA) encompasses all tumors originating in the epithelium of the bile ducts, including the intrahepatic bile ducts (ICCA) and extrahepatic bile ducts (ECCA). The incidence of ICCA and ECCA has increased in the last few decades, and molecular advances in both entities have brought understanding of their differences and allowed treatment advances aimed at personalized therapy. In this review, we discuss recent progress in the molecular landscape of CCAs, emerging treatment biomarker-guided strategies, and future insights into the management of advanced disease.

## Introduction

Intrahepatic cholangiocarcinoma (ICCA), extrahepatic cholangiocarcinoma (ECCA), and gallbladder cancer are designated as biliary tract cancers (BTCs). In 2019, in the United States, there was an estimated total of 54,390 new cases (liver cancer and BTC), and there were approximately 35,740 deaths due to these diseases in the same year^1,2^. The definition of an ICCA is a cholangiocarcinoma (CCA) detected inside the hepatic parenchyma, whereas ECCA is a type of tumor located outside the liver parenchyma. These tumors can arise in any portion of the extrahepatic bile duct and can be additionally classified as hilar or distal CCA^1^. In this review article, we discuss several reports that present the best evidence for the management of CCA and molecular insights of personalized approaches, including checkpoint inhibitors and fibroblast growth factor receptor (FGFR) inhibitors.

## Localized cholangiocarcinoma

The treatment for localized CCA is based on the complete resection of the primary tumor. Analysis of data from 535 patients with ICCA resected in a multi-institutional database revealed a median overall survival (OS) of 27.4 months. Most disease-specific cancer deaths (65.6%) occurred in the 24 months after surgery. Factors associated with worse survival include larger tumor size, multifocal disease, vascular invasion, lymph node metastasis, and advanced stage. One of the limitations of this study was the absence of data about the perioperative regimens used^3^. The recommendation for adjuvant chemotherapy in complete resected BTC is based on two randomized controlled trials. In the phase III trial BILCAP, 447 patients with BTCs were randomized after surgical resection to receive capecitabine or observation. In both groups, 38% of patients had a positive margin resection. Although the study did not reach statistical significance in the intention-to-treat analysis, in the pre-specified per-protocol analysis, the mean OS was 53 months (95% confidence interval [CI] 40 months to not reached) in the capecitabine group and 36 months (95% CI 30–44) in the observation group (adjusted hazard ratio [HR] 0.75, 95% CI 0.58–0.97; *P* = 0.028). These results encourage the use of adjuvant capecitabine as an option for patients with resected BTC^4^. A multicenter prospective randomized controlled phase III trial evaluated a combination of mitomycin C and fluorouracil in resected pancreatobiliary carcinomas. An unplanned subgroup analysis in gallbladder carcinoma suggested improvement in 5-year survival rate in the chemotherapy group (26.0%) compared with the control group (14.4%) (*P* = 0.0367). No differences were seen with 5-year survival among patients with CCA^5^.

Concurrent chemo-radiotherapy is often used in the perioperative strategy for ECCA. A phase II study with 79 resected BTC patients (SWOG S0809) treated with adjuvant chemotherapy (gemcitabine and capecitabine) and capecitabine plus radiotherapy provided data for this modality. With a manageable safety profile, the 2-year survival was 65%, with a median OS of 35 months^6^. For patients with unresectable hilar CCA, multimodal therapy with neoadjuvant chemotherapy and radiotherapy and liver transplant has emerged as a promising option, as described in findings from the Mayo Clinic and other groups^7–9^. A multicenter retrospective study of 216 patients with early stage unresectable peri-hilar CCA who were treated with neoadjuvant concurrent chemotherapy and radiotherapy followed by liver transplantation showed promising results after rigorous selection for the procedure. The overall recurrence-free survival rate in 5 years was 65%^10^. A retrospective comparison between hilar CCA patients treated with upfront resection with curative intent or neoadjuvant treatment followed by liver transplantation found better OS associated with the latter strategy, with 3-year survival rates of 72% versus 33% and 5-year survival rates of 64% versus 18%, *P* <0.001^11^. Important questions regarding liver transplantation should be addressed considering limited supply of liver allografts and the need for life-long immunosuppression^12^. Data for specific recommendations in the perioperative treatment of BTCs are limited because of the results and quality of the available trials; prospective studies should be designed to address resection strategies^1,12^. Gemcitabine plus oxaliplatin is not recommended in the adjuvant setting for resected BTCs based on recent negative randomized phase III trials^13,14^.

## Advanced disease

Chemotherapy is often used for the management of patients with metastatic CCA. The randomized phase III trials ABC-02 and ABC-06 provide results from chemotherapy regimens in the first line and second line of systemic treatment in BTC. In the first trial, ABC-02, 410 locally advanced or metastatic patients were randomized to receive cisplatin and gemcitabine or gemcitabine alone. After a median follow-up of 8.2 months, OS was improved with the addition of cisplatin to gemcitabine: median OS was 11.7 months in the cisplatin–gemcitabine group and 8.1 months in the gemcitabine-alone group (HR 0.64, 95% CI 0.52–0.80; *P* <0.001). The median progression-free survival was also improved with the combination: 8.0 months versus 5.0 months in the gemcitabine-only group (*P* <0.001)^15^. ABC-06 was a randomized phase III trial of the chemotherapy regimen modified fluorouracil plus oxaliplatin (FOLFOX) in the second-line setting for advanced BTC. Patients with disease progression on cisplatin and gemcitabine were randomized to either active symptom control (ASC) or ASC plus modified FOLFOX. From 27 March 2014 to 4 January 2018, 162 patients were randomized, 81 in each arm. Median OS and survival rates were improved with chemotherapy; median OS was 6.2 months with chemotherapy and 5.3 months with ASC alone. The 6-month and 12-month survival rates for chemotherapy plus ASC were 50.6% and 25.9%, respectively, and for ASC alone were 35.5% and 11.4%, respectively. Based on these results, modified FOLFOX could be considered a standard chemotherapy regimen for the treatment of patients who failed cisplatin plus gemcitabine in first-line systemic treatment^16^.

Lately, several studies have demonstrated potential targets for personalized treatment in BTCs^17^. Genomic profiling in large cohorts of patients with BTC aim to assist the stratification of patients to targeted therapy. Overall, the tumor mutational burden (TMB) in BTC is low; in an analysis of 803 patients with biliary cancers, the median TMB was 3.0 (0.8–6.1) Mut/Mb^18^. In another cohort with 239 cases, just 6% were considered high TMB, with the cutoff of 11.13 Mut/Mb^19^. The genes most frequently associated with genomic alterations are *TP53*, *KRAS*, *ARID1A*, *SMAD4*, *CCND1*, *MET*, *MDM2*, *CDKN2A*, and *CDKN2B*, and the most common actionable gene targets are *FGFR2* fusions, *IDH1* mutations, and *HER-2* and *MET* amplifications; actionable targets are commonly observed in ICCA^17,18^. It is estimated that between 13 and 17% of ICCAs harbor genomic alterations in the *FGFR2* gene and that most of these alterations (i.e. fusions) predict tumor sensitivity to anti-FGFR2 tyrosine kinase inhibitors^17,18^. BJG 398 (infigratinib), a pan-FGFR kinase inhibitor, was first evaluated in a phase II study in patients with *FGFR* genomic alterations, and *FGFR2* fusions were detected in 48 (78.7%) patients. In this study, 61 patients were treated. The overall response rate was 14.8% and disease control rate was 75.4%. In this subgroup of chemotherapy-refractory patients, the responders were restricted to cases with *FGFR2* fusions^20^. Another pan-FGFR kinase inhibitor, pemigatinib, was similarly evaluated in a phase II trial. Patients who had disease progression to at least one systemic treatment received oral pemigatinib. The primary endpoint was response rate, and other outcomes were estimated including OS and safety. There were three cohorts of patients in this study: cohort A included patients with *FGFR* gene rearrangements/fusions. Of 107 patients (cohort A), the overall response rate was 35.5% (95% CI 26.5–45.4%), disease control rate was 82%, and median OS was 21.1 months. The most common treatment-related adverse event was hyperphosphatemia (60%)^21^. Infigratinib and pemigatinib are being further evaluated in randomized phase III trials in previously untreated advanced BTC (NCT03773302, NCT03656536). Despite the encouraging results of these molecules, emerging mutations and acquired resistance have been observed in several cases after exposure to both drugs^17,22–24^. TAS 120 is an irreversible FGFR1–4 inhibitor and was the first to be evaluated in a group of 45 BTC patients harboring FGFR aberrations. In this study, 13 patients had previously received a reversible FGFR inhibitor. The overall response rate was 25%, and four patients previously treated with an FGFR inhibitor had a partial clinical response^25^. The efficacy of TAS 120 in acquired FGFR mutations after FGFR reversible inhibitors in ICCA was evaluated and confirmed in cell line models, but limited activity against some acquired mutations after infigratinib exposure including V565F was observed^22^. Interestingly, Debio-1347, another pan-FGFR inhibitor, remained active against this specific mutation^22,26^. Other pan-FGFR kinase inhibitors are being evaluated in ICCA with *FGFR* fusions, including erdafitinib and derazantinib; both drugs demonstrated anti-tumor activity and a tolerable safety profile in phase I/II trials^27,28^. With all of these drugs targeting FGFR, a new understanding of the relationship between the structure of inhibitory molecules of FGFR and the acquisition of resistant mutations will be necessary for the development of future studies in the management of these patients.

One of the most common driver genetic alterations in ICCA is gain-of-function mutations in the isocitrate dehydrogenase (IDH)-1 enzyme, observed in 20–25% of ICCA patients^29,30^. Ivosidenib, a targeted inhibitor of mutant IDH1, was initially evaluated in 73 patients with mutant advanced CCA, demonstrating a tolerable safety profile^31^. Furthermore, the drug was compared with placebo in a randomized phase III trial of patients previously treated with chemotherapy and who had disease progression with the treatment. ClarIDHy is a randomized phase III trial in metastatic IDH1 mutant CCA. In this trial, 185 patients were randomized to ivosidenib or placebo in a 2:1 fashion. In this group of patients, 46% had two prior systemic treatments. The primary end-point of progression-free survival was met; the median progression-free survival for ivosidenib was 2.7 months and for placebo was 1.4 months (HR 0.37, 95% CI 0.25–0.54; *P* <0.0001). OS with ivosidenib was not statistically significantly different to the placebo arm. However, in this trial, 57% of placebo patients crossed over to ivosidenib. Ivosidenib is not currently approved by the US Food and Drug Administration (FDA) for the treatment of IDH1 mutant advanced CCA^32^. BAY 1436032 is another oral IDH1 inhibitor that is being evaluated currently in advanced solid tumors (NCT02746081).

Pembrolizumab is an anti-programmed cell death protein 1 (PD-1) antibody that was evaluated in mismatch repair-deficient tumors and demonstrated clinical benefit in a large subset of gastrointestinal malignancies, ultimately being approved by the FDA as an agnostic treatment for microsatellite instability-high (MSI-H) tumors^33,34^. Unfortunately, the MSI-H phenotype is not very common in BTC, and its incidence ranges from 5–10%^35^. From a total of 11 patients with advanced mismatch repair-deficient BTC enrolled in these trials treated with pembrolizumab, the response rate was 27%, with duration of response ranging between 11 and 20 months^36^. The prevalence of programmed death-ligand 1 (PD-L1) in BTC is similar to MSI and ranges from 5–10%^37,38^. Combined analysis from two cohorts of advanced BTC patients treated with pembrolizumab provided data for antitumor activity and biomarker selection. In these studies (Keynote 028 and Keynote 158), patients who had failed at least one systemic treatment (no previous immunotherapy allowed), with measurable disease and Eastern Cooperative Oncology Group (ECOG) performance status of ≤1, were treated with pembrolizumab. PD-L1 positivity (membranous PD-L1 expression in ≥1% of tumor and associated inflammatory cells or positive staining in stroma) was not necessary for the Keynote 158 study. All of the 24 patients in Keynote 028 and 61 out of 104 in Keynote 158 had PD-L1 positivity. The response rate ranged from 5.8% in Keynote 158 to 13% in Keynote 028. No greater effectiveness of pembrolizumab was observed in the PD-L1-positive group of patients^39^. Nivolumab, an another anti-PD1 antibody, showed activity in previously treated BTC in a phase II trial. In this multi-institutional study, a total of 54 patients previously treated with at least one but no more than three lines of systemic treatment were evaluated. The median OS was 14.24 months (95% CI 5.98 months to not reached). PD-L1 expression was associated with prolonged progression-free survival (HR 0.23, 95% CI 0.10–0.51; *P* <0.001)^40^. In order to enhance the activity of checkpoint inhibitors in BTC, randomized trials addressing checkpoint inhibitors combined with chemotherapy and other target drugs in first- and second-line therapy of systemic treatment in advanced BTC are underway (NCT03639935, NCT03101566, NCT03110328, NCT03260712, and NCT04003636).

## Conclusion

New trials evaluating combinations of checkpoint inhibitors with chemotherapy in advanced CCA could enhance the response rate and outcomes of immunotherapy alone. FGFR2 inhibitors are being evaluated in the first-line treatment of patients with *FGFR2* fused advanced BTC, and these trials could change the landscape of systemic treatment of CCA in the next few years.
